# Two complementary approaches for efficient isolation of Sertoli cells for transcriptomic analysis

**DOI:** 10.3389/fcell.2022.972017

**Published:** 2022-09-06

**Authors:** Jana Petrusová, Jasper Manning, Jan Kubovčiak, Michal Kolář, Dominik Filipp

**Affiliations:** ^1^ Laboratory of Immunobiology, Institute of Molecular Genetics of the Czech Academy of Sciences, Prague, Czechia; ^2^ Laboratory of Genomics and Bioinformatics, Institute of Molecular Genetics of the Czech Academy of Sciences, Prague, Czechia

**Keywords:** Sertoli cell, flow cytometry, FSHr, Occludin, spermatogenesis

## Abstract

Sertoli cells (SCs) are the only somatic cells that reside in seminiferous tubules of testis. They directly interact with and support the development of germ cells, thus have an indispensable role in the process of spermatogenesis. SCs first appear in a proliferative state and then, with the initiation of the first wave of spermatogenesis, progress to a mature “nurturing” state which supports lifelong continuous sperm production. During this development, the SC transcriptome must adapt rapidly as obstacles in SC maturation often result in deficiencies in male fertility. Due to its importance in spermatogenesis, a reliable, rapid, and precise method for the isolation of high purity, viable and unadulterated SC has been largely missing. We have developed an improved method for the preparation of a testicular single cell suspension comprised of two alternative protocols to separate SCs from the rest of the testicular cells by FACS. The first sorting scheme is based on their co-expression of surface specific markers, FSHr and Occludin-1, while the second focuses on the co-staining of SCs with FSHr-specific antibody and Hoechst 33342, which discriminates DNA content of testicular cells. The entire procedure can be completed in less than 3 h which permits the analysis of the development-related transcriptional profile of these cells. Notably, our comparative study showed that this method resulted in a SC transcriptome that is largely comparable to SCs which were briskly isolated due to their cell-specific expression of fluorescent protein. Interestingly, we also show that SCs sorted as FSHr^+^Occludin^+^ cells contained a tangible portion of transcripts from all types of testicular germ cells. Sorting of SCs according to their 2C DNA content significantly reduced the presence of these transcripts, thus seems to be the most suitable approach for accurate determination of the SC transcriptome. We believe that these novel approaches for the isolation of SCs will assist researchers in the elucidation of their function as well as their role in spermatogenesis and disorders related to male infertility.

## Introduction

Sertoli cells play a vital role in the process of spermatogenesis, a phenomenon which is comprised of a chronological series of cellular events that result in the production of sperm. Spermatogenesis occurs within the seminiferous tubules (STs), the functional unit of mammalian testis. STs contain two types of cells, Sertoli cells (SCs) and germ cells (GCs). SCs are polarized, columnal somatic cells that form the inner circumference of STs with each cell extending from the base of the tubule to the lumen (Dunleavy et al., 2019). Within the base of the tubule, SCs intermingle with spermatogonial stem cells (SSCs) which initiate the development of sperm early after birth, postnatal day (PND) 9 (Bellve et al., 1977b; Kluin et al., 1982; de Rooij, 1998). SSCs undergo the first (primary spermatocytes) and second (secondary spermatocytes) meiotic division which give rise to haploid spermatids and ultimately lead to the production of mature sperm. SCs are an intriguing component of STs because their unique three-dimensional morphology (Franca et al., 2016) endows them with the capacity to create a distinctive nursing environment for the development and maturation of GCs to which they are strongly attached *via* tight junctions (Griswold, 2018). SCs themselves undergo a two–stage developmental program. During embryonic development and early perinatal life, SCs proliferate to build up the structure and volume of STs. Then, during male puberty (between day 14 and 30 in mice) SCs acquire two new critical attributes: 1) a nursing function that fully supports the process of spermatogenesis, which includes phagocytosis of residual cytoplasm released from elongated spermatids during spermiation and 2) a barrier function designated as the blood-testis barrier (BTB). BTB is physically demarcated by basal ectoplasmic specialization structures which contain junction proteins such as Occludin, Claudin, and ZO-1 which are situated within the membranes, interlocking neighboring SCs, stabilizing the barrier structure (Yan and Cheng, 2005; Lie et al., 2006). Importantly, during this transition period, SC proliferation ceases and extensively modulates its gene expression profile to support nourishing and BTB functions (Zimmermann et al., 2015).

It is clear that a comprehensive understanding of the role of SCs in spermatogenesis requires specific methodological tools to isolate a pure population of SCs. Thus far, one of the most effective approaches for their isolation and/or enrichment is the use of a transgenic mouse strain with SOX9-driven expression of eGFP in SCs (Zimmermann et al., 2015). This approach, however, might not be affordable for all labs, especially if it is necessary to perform crosses with other genetically modified strains. Alternative approaches which use standard mouse strains are, due to a lengthy cultivation step that is required to enrich the SC population by adhesion to a plastic dish, time-consuming and requires many animals. To illustrate this, the protocol reported by Lakpour et al., which is based on the cultivation of a ST suspension on lectin pre-coated dishes requires 3–5 days followed by flow cytometric (FACS) sorting of SCs according to their FSHr positivity. It is of note that although the authors reported a high purity of FACS-sorted SCs, the specific sorting strategy and post-FACS analysis of the resulting SC population was not provided (Lakpour et al., 2017). An analogous protocol for the isolation of rat SCs and establishment of a primary culture, which is just as labor intensive, requires at least 24 h and 10 male rats (Bhushan et al., 2016). An alternative procedure which relies on the enzymatic digestion of STs with subsequent centrifugal elutriation in a Percoll gradient, which isolates specific testicular cell populations with 80–95% purity (Chang et al., 2011), requires between 5–7 h to complete. In general, all protocols for SCs isolation reported so far, apart from the Zimmerman protocol, are time consuming and yield a less pure population of SCs. It is of note, that only the Zimmerman protocol has been used for bulk-RNA sequencing and subsequent qRT PCR analyses of gene expression in SCs. Given that the lengthy time period needed for the preparation of SCs can induce undesirable changes in the gene expression profile and survival of SCs, reducing this time is critical for accurate analysis of the SC transcriptomic profile during development.

In this study, we aimed to develop an alternative protocol for the preparation of an optimal testicular single cell suspension in which we can unambiguously identify and isolate SCs by antibody staining by flow cytometry. This approach takes less than 2 h and the resulting isolated SCs are comparable to those obtained using the current method of SC isolation of sorting SCs from the SOX9-eGFP mouse strain. We believe that this method can be easily implemented in any laboratory that has access FACS and most importantly doesn’t require the handling of genetically modified mice. We also show that in addition to classical FACS-sorting of antibody stained SCs, which show a relatively high level of “contamination” with transcripts from all types of GCs, SCs can be sorted as somatic cells with 2C DNA content. This approach decreases this contamination by 10-100-fold which allows for a more accurate assessment of the transcriptional landscape of SCs.

## Materials and equipment

### Experimental animals and tissue collection

All mice used were on C57B6 genetic background and were housed under standard SPF conditions at the mouse facility of the Institute of Molecular Genetics in Prague, Czech Republic. The pups were euthanized on PND7, PND14, PND21, PND28, PND35, and PND42 to study spermatogenesis. Immediately after euthanasia by cervical dislocation, testes were removed, dissected, decapsulated, and processed to ST and ultimately a single cell suspension was prepared.

### Single cell suspension from mouse testes

This protocol is adjusted for testicular tissue which is processed in a 15 ml conical tube. Notably, decapsulated testes from both testes extracted from newborns up to 3-week-old males can be processed in the same tube, while from older males, each testicle should be processed in a separate tube. Enriched Krebs-Ringer bicarbonate medium (EKRB) was used for a single cell suspension preparation. EKRB is freshly prepared from a 10x concentrated KRB stock (which had not been frozen for more than 3 months) and supplemented with Sodium d-Lactate (Sigma-Aldrich), Sodium Pyruvate (Gibco), NaHCO_3_ and CaCl_2_ (Penta Chemicals Unlimited) immediately before the start of experiment (EKRB composition and final concentrations of components are listed in Supplementary Chart S1). ST from decapsulated testicles were immediately washed in 5 ml of EKRB and mechanically disrupted by vigorous shaking by hand for at least 10 s. Subsequently, 10 μL of Collagenase-D from a 50 mg/ml *Clostridium histolyticum* (Roche) stock and 5 μL of 10.000 U/ml DNase I (Sigma Aldrich) is added. It is important not to shake the sample after DNase I has been added since the cells become more sensitive to mechanical damage. Then, samples were incubated for 10 min in a water bath at a physiologically relevant temperature (i.e., for samples derived from animals ≤ PND14 at 37°C, for those ≥ PND15 at 32°C) with periodic tube inversion to maintain STs in suspension. STs were then disrupted using a 10 ml serological pipet, pipetting 10–15 times until short tubular fragments remain. Next, fresh pre-warmed EKRB + Collagenase + DNase cocktail is again added followed by a 10-min incubation in a water bath with periodic tube inversion. After incubation, the sample is again subjected to pipetting with a 10 ml serological pipet 10–15 times to disrupt the remaining short fragments of STs ending with an opaque suspension with very few or no ST fragments visible. This rough single cell suspension was immediately filtered through a 100 μm strainer allowing SCs to flow through into a 50 ml conical tube. The cells were then pelleted by centrifugation (Benchtop Eppendorf centrifuge 5810 R equipped with a swing-out rotor), at 300 g for 8 min. The supernatant is discarded and the cell pellet is resuspended in 200 μL of EKRB by gentle tapping at the tube proceeded by immunolabelling. Testicular interstitial cells were isolated from decapsulated testes by vigorously shaking for 1 minute in EKRB buffer. The STs were allowed to settle and the supernatant was taken and used as the single cell suspension of interstitial cells. These cells were sedimented by centrifugation (300 g for 10 min) and consequently resuspended in Hanks’ Balanced Salt solution (HBSS, Gibco) with 0.1% EDTA and 1% BSA (both Sigma-Aldrich) and proceeded by immunolabelling.

### Immunodetection of SCs surface antigens and spermatocytes population analysis

To identify surface markers, we used an antibody specific to the Follicular stimulating hormone receptor (FSHr) conjugated with Phycoerythrin, diluted 200x (FSHr-PE, Bioss, bs-20658R-PE) and Occludin-Alexa 488, diluted 200x (Signalway Antibody, C45047). Cells were incubated in the dark at either 32°C or 37°C for 1 h. The cells were then diluted with an additional 1 ml of pre-warmed EKRB prior to FACS analysis. It is important to avoid any centrifugation or agitation before flow cytometry analysis. Hoechst 33258 (Invitrogen, 10 mg/ml solution in water) was added to a final concentration of 10 μg/ml to discriminate live from dead cells. Interstitial cells were isolated mechanically by vigorous shaking of decapsulated testes in EKRB. After sedimentation of freed STs, the supernatant was collected and used for staining with FSHr-PE and Occludin-488, along with conjugated immune cell markers CD45-Cy7, MHCII-APC, and CD11b-PerCP-Cy5.5, each diluted 200x (all from BioLegends).

To identify FSHr^+^ SCs that have engulfed primary spermatocytes or spermatocytes attached to its surface, we took advantage of a well-established method of Hoechst-based FACS analysis of a heterogenous cell population in ST (Lassalle et al., 2004; Bastos et al., 2005; Gaysinskaya et al., 2014). Using this method, the ST cell suspension revealed a “scythe-like” profile, where somatic cells (SCs and SSC—2n), GCs (i.e., primary spermatocytes—4n and secondary spermatocytes—2n) and round spermatids and sperm (n), appear as individual populations. Towards this end, a single cell suspension was incubated for 30 min, in the dark at 32°C or 37°C, with FSHr-PE antibody, followed by incubation with Hoechst 33342 (Invitrogen, 10 mg/ml solution in water) at the final concentration 20 μg/ml for another 30 min. Prior to FACS analysis, the sample was placed on ice to keep cells from succumbing to rapid death due to the Hoechst toxicity. For Hoechst visualization and FACS analysis, we employed standard measurements which have been previously utilized (Gaysinskaya et al., 2014). Briefly, Hoechst was excited using a 375 nm laser, and was detected in two distinct channels: the “Ho Blue” (450/40 nm band-pass filter) and the “Ho Red” (670 nm long pass filter).

### Technical specifications and sorting conditions for FACS sorting of SCs

For all FACS-sorting experiments, we used Influx (BD Biosciences), the polychromatic high-speed jet-in-air sorter, which is equipped with five solid state lasers (355, 405, 488, 561, and 640 nm), forward (FSC) and side (SSC) scatter detectors, and 14 fluorescence detectors. Aerosols from the sort chamber were cleared by an aerosol evacuation system. The entire sorter is enclosed in a class II. biological safety cabinet. Cells were sorted through a 100 μm nozzle using the following sort settings: Piezo Amplitude 0.95, drop frequency 40 kHz, with two-drops pure sorting quality. In 60 min of FACS-sorting, we obtained between 5.000–10.000 of SCs, depending on the sample density and quality. Using these sorting parameters, the post-sort analysis of FSHr^+^Occludin^+^ SCs showed 96% viability with all cells being positive for the sorting markers (Supplementary Figure S1B). Sorter is supported by BD FACS software (BD Biosciences). Flow cytometric analysis was performed on a FACSymphony (BD Biosciences) flow cytometer, equipped with six solid state lasers (355, 405, 447, 488, 561, and 637 nm) and BD FACSDiva software. The analysis of collected data was executed using FlowJo™ v10.8 Software (BD Life Sciences).

### Immunofluorescence protein detection, SC cultivation and microscopy

To observe protein immunofluorescence and successfully cultivate the SC population, FACS- sorted cells were placed onto either a slide for immune-fluorescence microscopy or in a Petri dish containing Dulbecco′s Modified Eagle′s Medium (DMEM, Gibco) supplemented with an antibiotic cocktail containing penicillin (working solution 100 IU/ml) and streptomycin (working solution 100 μg/ml). The cells were placed on Super-frost glass slides (Thermo Scientific™ SuperFrost™ Microscope Slides) and covered with freshly made 4% formaldehyde (Sigma-Aldrich). The cells were fixed at ambient temperature for 20 min. The slides were then placed in a laminar flow hood for 10 min to allow partial evaporation and attachment of the cells to the slide. The slides were then immersed in 0.1% Triton-X100 in PBS and permeabilized for 20 min. Next, the slides were washed in 1x PBS for 5 min, transferred to 1% BSA in PBS-T (0.1% Tween in PBS) and incubated at ambient temperature for 1 hour. The following antibodies were used and diluted in PBS-T: 200x FSHr-PE (Bioss, bs-20658R-PE), 200x SOX9 (Thermo Fisher Scientific, ABIN5950738). In the case of SOX9, goat-anti mouse secondary antibody, conjugated with Alexa 488 fluorophore, diluted 1000x was used. Samples were visualized with a conventional DeltaVision wide field microscope equipped with a U PLAN FL 20x/0.50 dry objective, Lumencore LED illumination, and a Photometrics CoolSNAP HQ high-sensitive CCD camera. All images were deconvolved with Huygens Professional, version 19.04 (Scientific Volume Imaging, Netherlands, http://svi.nl) and processed in Fiji ImageJ (Rasband, 1997).

### Isolation of mRNA and qPCR

Total RNA from FACS-sorted cells was extracted using a RNeasy Plus Micro Kit (Qiagen), reverse transcribed with RevertAid (ThermoFisher) transcriptase and random hexamers (ThermoFisher). Quantitative RT-PCR (RTqPCR) was performed using LightCycler 480 SYBR Green I Master mix (Roche) on a LightCycler 480 II (Roche). Each sample was tested in triplicate. Threshold cycles were calculated using LightCycler 480 1.5 software. Gene expression was calculated by relative quantification using mRNA levels of the housekeeping gene, CASC3, as a control (Pfaffl, 2001). Primers were designed using Primer-BLAST (NCBI, NIH). Primer sequences are listed in Supplementary Chart S2.

### RNA sequencing and analysis

SCs were sorted according to the protocol described above and RNA was extracted using a RNeasy Plus Micro Kit (Qiagen). cDNA synthesis, ligation of sequencing adaptors and indexes, ribosomal cDNA depletion, final PCR amplification, and product purification were prepared with a SMARTer^®^ Stranded Total RNA-Seq—Pico Input Mammalian library preparation kit v2 (Takara). Library size distribution was evaluated on an Agilent 2100 Bioanalyzer using the High Sensitivity DNA Kit (Agilent). Libraries were sequenced on an Illumina NextSeq 500 instrument using a 76 bp single-end high-output configuration resulting in ∼30 million reads per sample. Read quality was assessed by FastQC (0.11.9) and the sequencing adaptors were removed using Trim Galore! (0.4.5). The expression quantification at gene level was performed using Salmon (0.14.1) against the GRCm38 reference genome (ensEMBL assembly version 98). The sequencing data has been deposited into ArrayExpress database under the accession number E-MTAB-11895. The obtained data was compared to RNA sequencing analysis of SCs compiled by Zimmermann et al. (accession number GSE59698) (Zimmermann et al., 2015): the reads for the respective experiment were downloaded from the SRA archive (https://www.ncbi.nlm.nih.gov/sra) using the SRA toolkit and processed as described above. For both datasets, the transcripts per million (TPM) values were used for the downstream analyses and their values were variance stabilized by base two log-transformation. To mitigate technical differences between the experiments, we used ComBat and quantile normalization of the gene expression data to remove the induced batch effects. The principal component analysis (PCA) was performed using the ade4 package (4_1.7–19).

To visualize data similarity, a heatmap with previously described markers was plotted, namely: Plzf (Zbtb16), Sall4 and Sohlh1 for spermatogonia; Meioc, Prdm9, Top2a and Smc3 for early spermatocytes; Sycp1, Sycp2, Sycp3, and Hzafx (H2afx) for spermatocytes; Acrv1, Catsper3, Catsper4, Spata25, and Izumo1 for round spermatids; Prm3, Izumo2, and Tssk6 for elongated spermatids, and Wt1, Mro, Sox9, Amh, Dhh, Cst9, Rhox5, Fshr, Gata1, Ar, Cdkn1b, and Clu for Sertoli cells. All analyses were performed in the R statistical environment (4.2.1).

## Results

### Preparation of single cell suspension from mouse seminiferous tubules

**FIGURE 1:**
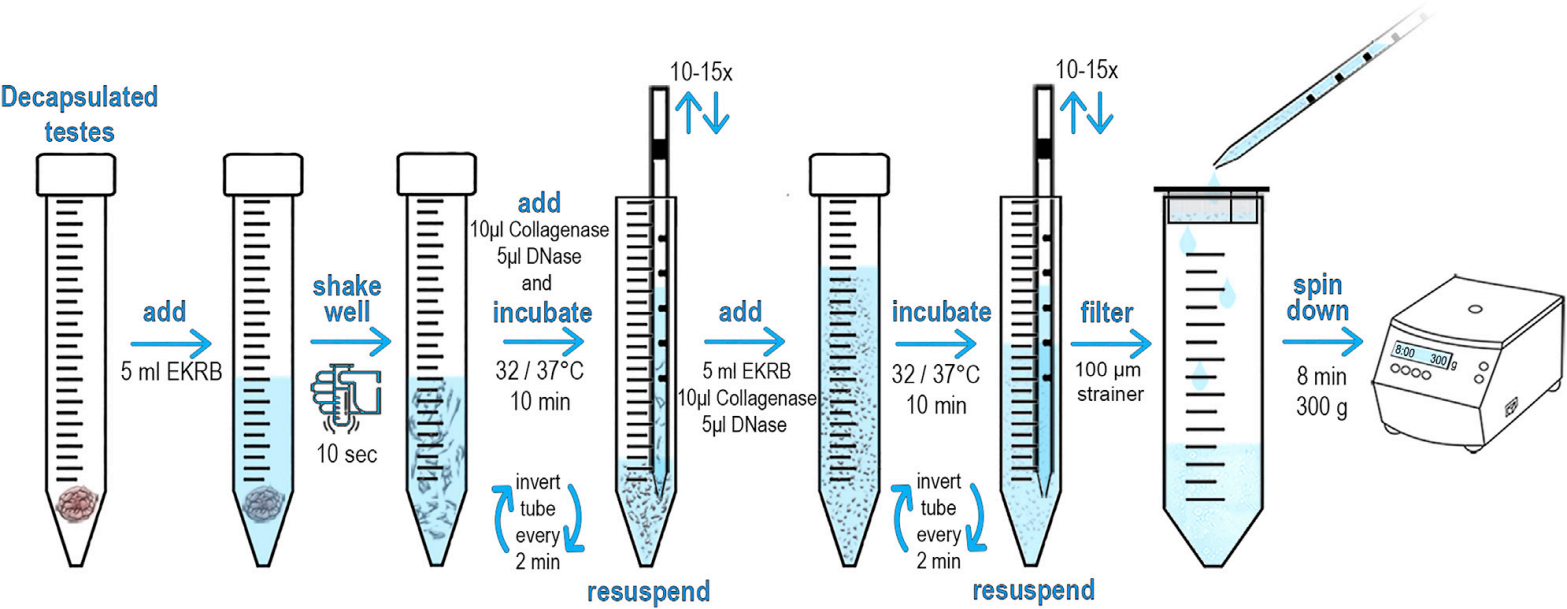
Schematic diagram and protocol of preparation of testicular single cell suspension. The male mouse was euthanized. An incision was them made under scrotum, testes were removed, dissected and transferred to a Petri dish. Using scissors, the *tunica albuginea* was opened to release the seminiferous tubules (STs). STs were immediately placed into 15 ml conical tube containing 5 ml of freshly made pre-warmed EKRB. The tissue was disrupted by vigorously shaking by hand for at least 10 s 10 μL Collagenase (50 mg/ml) and 5 μL of DNase (10.000U) was then immediately added and incubated at 32°C/37°C (according to age of the male) for 10 min. The samples were inverted every 2 minutes to keep the tubules in suspension. STs are then disrupted by slowly pipetting through serological pipet. Repeat 10–15 times until STs are fragmented. An additional 5 ml of EKRB, 10 μL Collagenase (50 mg/ml) and 5 μL of DNase (10.000U) is added and incubated for another 10 min at the desired temperature. The samples were inverted every 2 minutes to keep the tubules in suspension. The remaining ST fragments are disrupted by slowly pipetting through a serological pipet. Pipet up and down at least 10–15 times until the suspension appears opaque and the ST fragments are no longer visible. Transfer the cell suspension dropwise with serological pipet into a new conical tube that is equipped with an inserted 100 μm strainer to obtain a ST single cell suspension. Centrifuge the cell suspension for 8 min at 300 g to pellet the cells. Discard the supernatant and add 200 μL of pre-warmed EKRB. Gently re-suspend pelleted cells by tapping on the tube bottom. Add the conjugated antibody and incubate at 32°C/37°C in a dark place.

We adapted and modified the protocol for the preparation of a single cell suspension from Bellvé et al. (Bellve et al., 1977a; Bellve et al., 1977b). The original method was designed for the identification and sorting of primary spermatocytes present in ST. Our main goal was to modify this protocol to rapidly prepare and isolate viable SCs. Briefly, dissected testes were decapsulated and obtained STs processed in EKRB medium (Supplementary Chart S1). The tubules were then disrupted and the resulting cell suspension filtered through a 100 μm strainer. The cells were collected by centrifugation, incubated with fluorescently-conjugated antibody, and subjected to FACS analysis. This method is rather straightforward and can be completed in less than 2 h. A detailed protocol is depicted in Figure 1.

### Flow cytometric identification of SCs by combination of two specific surface markers

**FIGURE 2:**
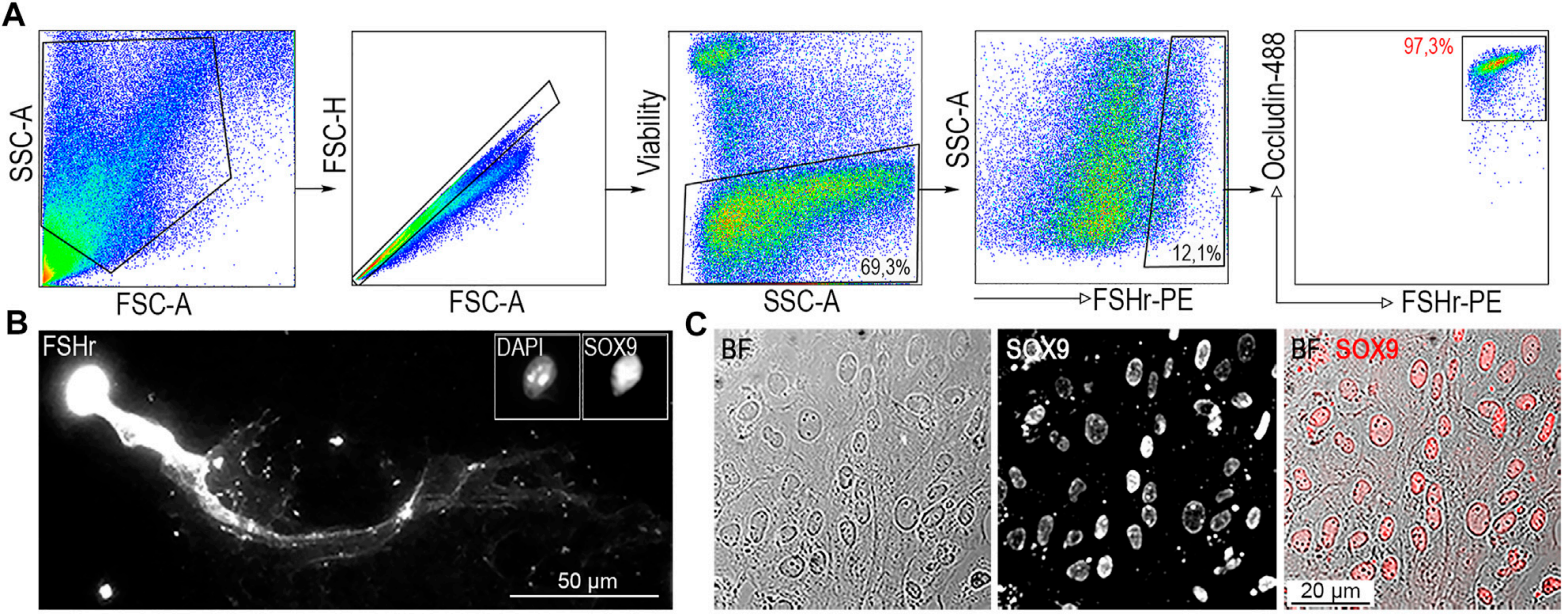
Identification of SCs from ST single cell suspension. **(A)** A single cell suspension generated from ST was incubated with FSHr-PE and Occludin-488 antibody. All FSHr^+^ cells positive for Occludin were sorted (right panel, red gate). Sorting gate contains 97,3% of cells. **(B)** To confirm specificity, double positive cells were analyzed by immunofluorescence microscopy to determine the presence of the transcription factor (TF), SOX9. **(C)** Immunofluorescence analysis of FSHr^+^Occludin^+^ double positive cell population cultured on a Petri dish confirming homogenity in the expression of SOX9 TF.

Using the method described in Figure 1, SCs from distinct phases of their developmental path in testes were purified. Fluorescently-conjugated antibodies against two SC markers - Follicular Stimulating Hormone receptor (FSHr) and Occludin were used to facilitate FACS-sorting of these cells. As shown in Figure 2A (and Supplementary Figure S1A), both proteins fulfilled the criteria of surface expression and cell-type specificity. FSHr is a plasma-membrane associated G-protein coupled receptor (Orth and Christensen, 1977; Kangasniemi et al., 1990; Simoni et al., 1997; McLachlan et al., 2002) whose expression in mouse SCs has been detected from E10.5 of embryonic development and onwards (Bailey et al., 2006). Along with Luteinizing hormone (LH) in Leydig cells, the expression of FSHr is regulated by the Hypothalamic-Pituitary-Gonadal (HPG) axis in response to the hypothalamic gonadotropin-releasing hormone (Oduwole et al., 2018). In contrast, Occludin, which is similar to Claudin-1, localizes to tight junctions which connect juxtaposing SCs in STs, thus represents a critical molecule in the maintenance of the blood–testis barrier (BTB) (Kaitu’u-Lino et al., 2007). Occludin transcripts appear in embryonal SCs at E14 *in utero*, however, its surface expression can be indicative of the maturation stage of SCs, since its increase correlates with the switch from juvenile (proliferative) to the mature (nursing) state of SCs (Fijak and Meinhardt, 2006; O'Bryan and Hedger, 2008). To justify the utility of Occludin as a surface expression marker on SCs, we measured the kinetics of expression in 1-week through 5-week-old males. As illustrated in Supplementary Figure S2A, Occludin surface expression levels gradually increased from the first week of life (Supplementary Figure S2A) with a markedly increased levels between week three and week five animals (Supplementary Figure S2B). In contrast, increases of FSHr surface expression on SCs, which have been observed from newborns to adult mice were not as dramatic (Supplementary Figure S2C). Thus, both FSHr and Occludin markers are deemed to by suitable for identification and isolation of SCs from newborns to adults.

### FSHr^+^ Occludin^+^ double positive population marks SC compartment of ST

To confirm the nature and determine the homogeneity of FACS-sorted FSHr^+^Occludin^+^ double positive SCs (FO-SCs) (Figure 2A), they were analyzed microscopically. Sorted cells were either fixed on a glass slide and immunostained or immunostained 72 h after seeding in culture. Those fixed onto the glass slide were immunoprobed for the presence of the transcription factor (TF), SOX9, which along with FSHr within ST is expressed exclusively by SCs (Harley et al., 2003; Sharpe et al., 2003). As shown in Figure 2B, when FO-SCs were re-stained and their anti-FSHr-labelled cytoplasmic membrane visualized, a “comet-like” morphology was observed with cell length exceeding 150 μm. This cell shape is a well-documented feature of isolated SCs (Chang et al., 2011). Importantly, the SCs were positively stained for SOX9. This result was corroborated using an adherent culture of FO-SCs which all exhibited positivity for SOX9 TF (Figure 2C). Therefore, the FSHr^+^Occludin^+^ subset of ST represents SOX9^+^ SCs, which remain viable after sorting, are suitable for further manipulation or culturing. While the FSHr staining alone distinguished SOX9^+^ SCs from the rest of ST cells (Figure 2A), the level of co-staining for Occludin evaluated the developmental stage of isolated SCs (Supplementary Figures S2A,B). It also allowed to remove approximately 2.7% of FSHr^+^ SCs with low or intermediate level of Occludin (Figure 2A, right panel). It is of note, cells from the interstitial space are all double negative for anti-FSHr/Occludin staining (Supplementary Figure S3), thus can not be included among FACS-sorted double positive FO-SCs.

**FIGURE 3:**
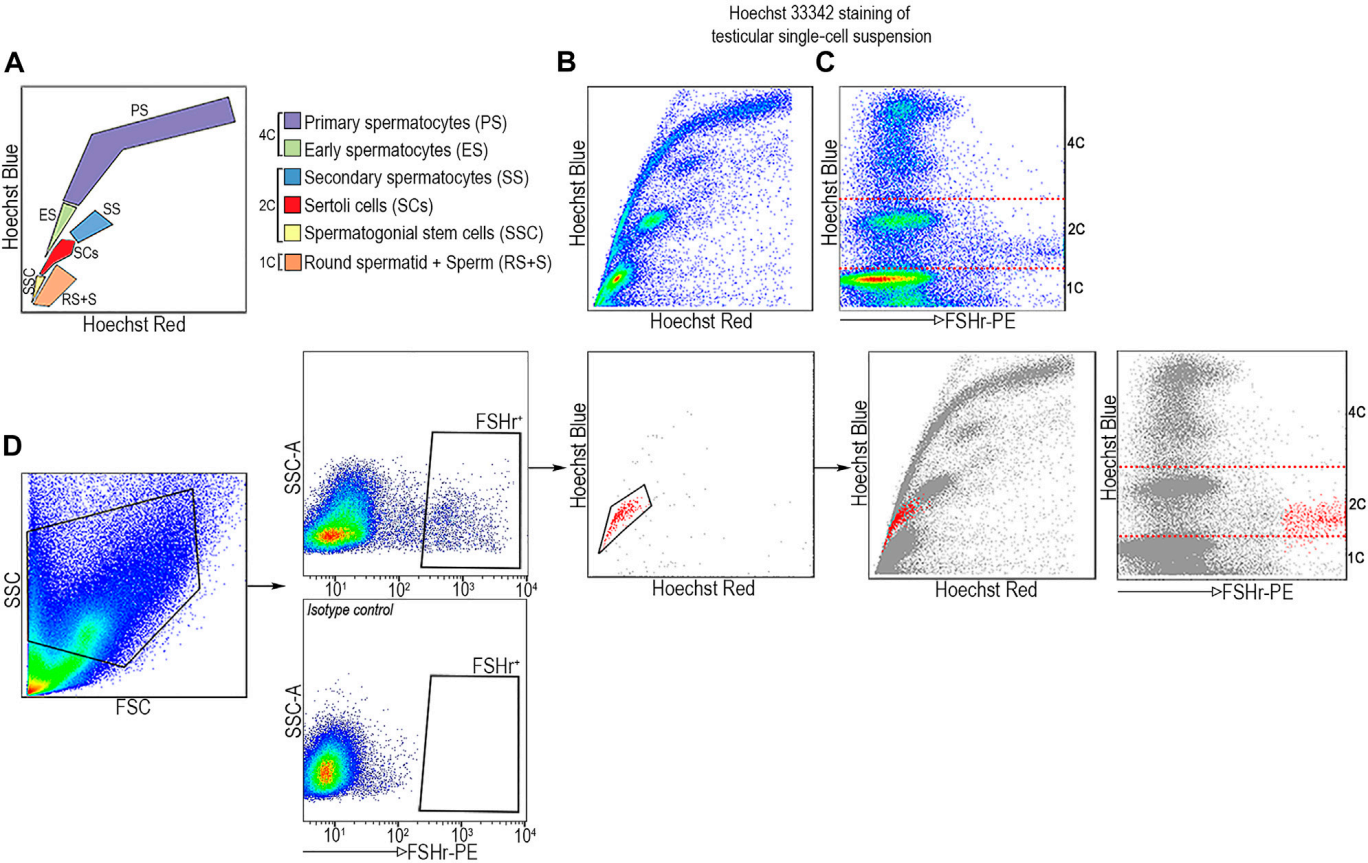
FSHr-PE positive cells from STs are found at position corresponding to SCs with 2C DNA content by Hoechst profiling. **(A)** Scheme of ST-derived cell type distribution after staining with Hoechst 33342. **(B)** Single cell suspension from STs with FSHr-PE staining plotted on Ho Red/Ho Blue profile. **(C)** FSHr^+^ cells are found in the area corresponding to 2C DNA content (Ho Blue) which is demarcated by the dashed line. **(D)** FSHr^+^ cells were plotted on Ho Red/Ho Blue profile and backgated to total ST cell populations. The areas with distinct content of DNA, 1, 2, and 4C, are indicated.

Since SCs are the only type of somatic cells in STs, we set out to determine if FSHr^+^ cells would display 2C DNA content by Hoechst profiling of all ST-derived cell-subsets. Using this approach, a testicular cell suspension from 5-week-old males was incubated with the DNA-binding dye, bis-benzamide Hoechst 33342 (Ho), followed by FACS analysis. Ho-staining allows one to separate cells based on their DNA content and chromosome structure (Watson et al., 1985; Bastos et al., 2005; Gaysinskaya et al., 2014) which relates to the chromatic shift in Hoechst emission from red to blue light spectra as the concentration of DNA-bound Hoechst dye increases (Petersen et al., 2004). Thus, Hoechst staining in the blue channel (Ho-blue), projected on the *y*-axis, separates distinct cell types which are present in ST into subsets with either 1C (round spermatid and sperm), 2C (SCs, SSCs, and secondary spermatocytes—SCII), or 4C (primary spermatocytes—SCI). Chromatin compaction of these subsets is projected on the *x* axis of the red channel (Ho Red). When combined, these two parameters provide a dot plot projection of a “scythe-like” shape in which each analyzed cell type occupies a predicted position (as depicted in Figures 3A,B). When FSHr^+^ cells from ST are backgated into the Ho Red/Ho Blue profile, they are overlaid at the position of SCs with 2C DNA content and a high FSHr signal (Figures 3C,D). This data further confirms that SCs can be unambiguously distinguished from other subsets of STs by the combination of its 2C DNA content and FSHr expression.

### Bulk RNA-seq confirmed the SCs population purity compared to other isolation protocols

Until this point, the simplest approach for isolation of the SC subset relied on the use of transgenic mouse expressing a SOX9-eGFP knock-in construct (Nel-Themaat et al., 2009). Due to the specificity of the SOX9-driven expression of eGFP in SCs (GFP-SC), the preparation and sorting of these cells is quick and straightforward (Zimmermann et al., 2015). This is of critical importance since it potentially decreases the effect of factors that can trigger undesirable changes in the vitality and gene expression of SCs. Since our modified method for the preparation of FO-SCs requires additional steps due to antibody staining, we assessed whether their expression profile would be comparable to GFP-SCs. Moreover, since both GFP-SC and FO-SCs were isolated at similar time points of their postnatal development (Zimmermann et al., 2015), we could perform a comparative analysis of their gene expression at juvenile (PND18-21), differentiating (PND25-28), and mature (PND35-70) stages of development.

**FIGURE 4:**
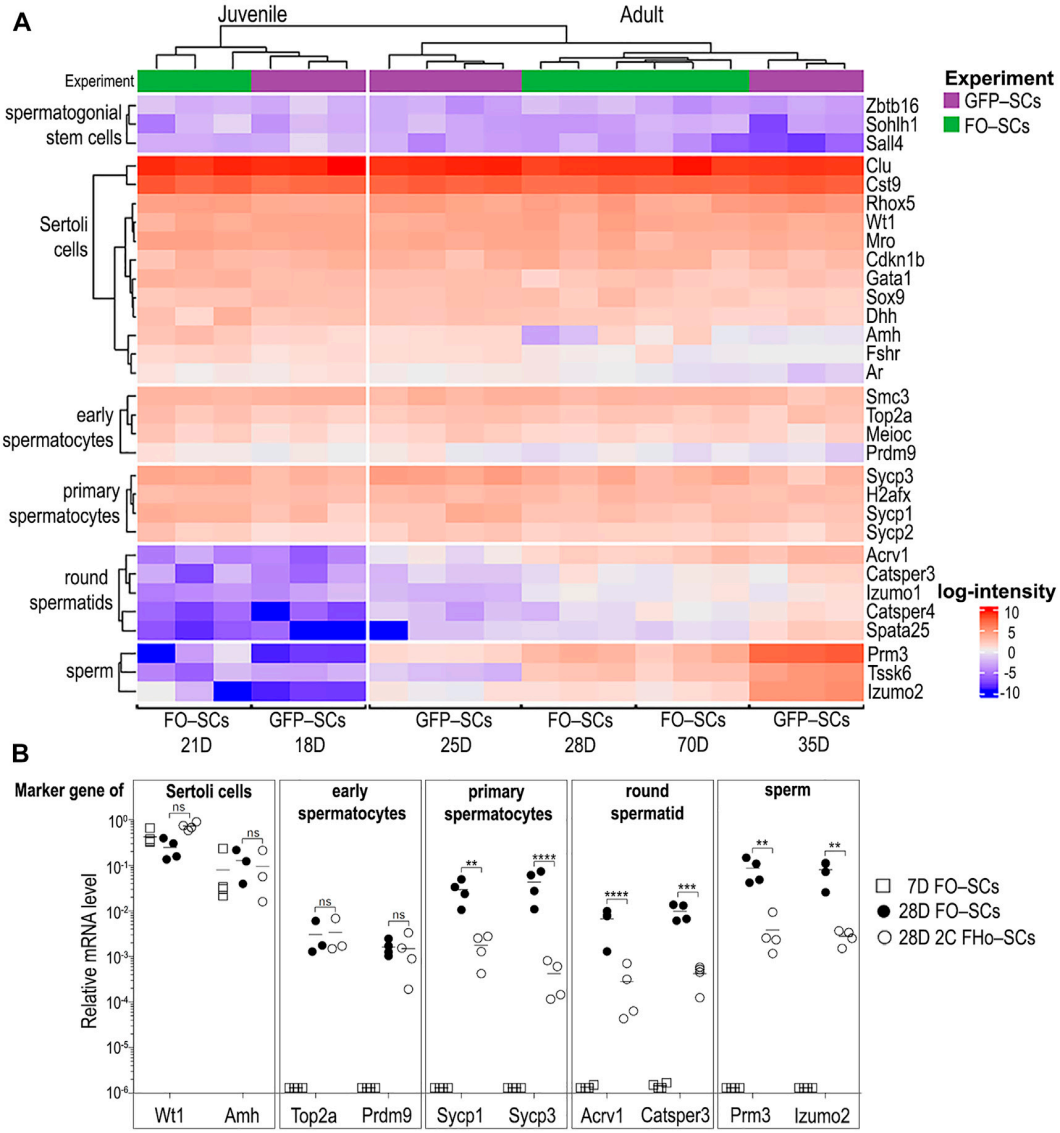
FACS-sorted SCs labelled with two surface markers exhibit the same gene expression profile as GFP expressing SCs. **(A)** The two sorting strategies showing SC transcriptome obtained by our method is comparable to that of transgenic GFP^+^ SCs (Zimmermann et al., 2015). In SCs, mRNAs from all types of GCs within STs was detected. **(B)** Selected signature genes of all ST cell types were quantified by qRT PCR in FACS-sorted FO-SCs as well as in FHo-SCs. Statistical evaluation of data was made by Unpaired *t*-test with the following *p*-values: Wt1 *p* = 0.8463; Amh *p* = 0.8744; Top2a *p* = 0.7986; Prdm9 *p* = 0.6578; Sycp1 = 0.0010; Sycp3 and Acrv1 *p* < 0.0001; Catsper3 *p* = 0.0004; Prm3 *p* = 0.0014; Izumo2 *p* = 0.0090.

Unsupervised clustering analysis showed that the expression profile of GFP-SCs and FO-SCs from a given developmental stage clustered together (Figure 4A and Supplementary Figure S4). This justifies the experimental suitability of the FO-SCs protocol and strongly supports the conclusion that the two protocols are equally efficient and suitable for transcriptomic analysis. It is of note that the expression profiles of 25–70-day old SCs are largely comparable. Perhaps, the only exception would be the slight downregulation of SC-specific genes, such as Amh (Anti-Müllerian hormone), FSHr, and Ar (Androgen receptor) that was observed at week five and later, which is consistent with previously published data (Lukas-Croisier et al., 2003; Edelsztein and Rey, 2019; Xu et al., 2019). However, as expected, marker genes of SSCs (Zbtb16, Sohlh1, and Sall4) were strongly suppressed in SCs (Figure 4A).

However, when we look at the specific marker genes of other types of male GCs i.e., early and primary spermatocytes, round spermatid and sperm, their expression in SC-FOs is surprising but also detectable in GFP-SCs (Figure 4A, discussed in Zimmermann et al., 2015). Specifically, marker gene transcripts of early and primary spermatocytes, which emerge in STs between PND9-14 and PND14–20, respectively, were already associated with juvenile SCs isolated from 18 to 21-day old testes. On the other hand, marker gene transcripts of round spermatid and sperm, which developmentally emerge in ST after PND21 (Zhu et al., 2015), were absent in SCs from 18 to 21 day old testes (Figure 4A, juvenile SC) and were detectable only after PND25 (Figure 4A, adult SC). The presence of these GC-subset marker gene transcripts in SCs that kinetically correlate to the appearance of these subsets in the ST offers two possible explanations. Owing to a strong molecular inter-connectivity of GCs and SCs during their differentiation and maturation in STs, intact GCs could stay physically attached to FO-SCs/GFP-SCs during FACS-sorting and “contaminate” the pool of SC-specific transcripts. Alternatively, since SCs absorb apoptotic GCs and/or actively recycle the cytoplasmic leftovers of GCs during spermiation, the contaminating RNAs from GCs would become inseparable from the pool of genuine SC transcripts. To decide, which of these two scenarios would prevail, SCs were FACS-sorted according to their FSHr^+^/Ho Blue profile (FHo-SC, as described in Figures 3C,D). Since the Ho Blue profile ensured that only FSHr^+^ SCs with 2C DNA content were sorted, the presence of surface-contaminating GCs would be eliminated. We compared the presence of selected marker gene transcripts in FO-SCs from 28-day-old males which were FACS-sorted either using FSHr^+^/Occludin^+^ or FSHr^+^/Ho Blue staining. FACS-sorted FO-SCs from 7-day-old mice were used as a control. Using RT-qPCR, genes specific for SCs (Wt1, Amh) were detected, as well as those for early spermatocytes (Top2a, Prdm9), primary spermatocytes (Sycp1, Sycp3), and round spermatids and sperm (Acrv1, Catsper3, Prm3, Izumo2) (Figure 4B). Regardless of the method used for the isolation of SCs, their marker genes (Wt1 and Amh) were expressed at comparable levels in all three samples tested. Importantly, the expression of all GC marker genes in SCs from 7-day-old testes were undetected or observed at negligeable levels, thus confirming that in the absence of spermatogenesis, the transcripts of these genes were not present in SCs. Except for the specific marker genes of early spermatocytes, SCs sorted according to FSHr^+^/Ho Blue profile showed 10–100-times diminished transcripts levels of all specific gene markers of the main types of GCs (Figure 4B). This demonstrated that the RNA contamination of SCs came from physically associated GCs. However, extremely low but reproducible levels of RNA contamination likely came from recycled and internalized parts of GCs which would make them inseparable from SCs by FACS. It seems that in the case of early spermatocytes, this type of contamination is predominant because FSHr^+^/Ho Blue profile sorting of SCs failed to decrease the levels of their specific marker gene transcripts. This is consistent with the observation that the function of SCs associated with the internalization of apoptotic GCs starts during the first wave of spermatogenesis (PND9-30) and persists over the lifetime of the cell (Geyer, 2017).

## Discussion

The efficiency of our method for isolation of SCs is based on strict adherence to a rule of the 3Ts: Temperature, Time and Tenderness.

Temperature. The temperature for sample incubation is critical for SC viability. Since newborns and juvenile male mice (pre PND14) testicles are still in the abdominal cavity (Hadziselimovic and Herzog, 1993; Hutson et al., 2010), the optimal incubation temperature must be physiological body temperature i.e., 37°C. Once the testes have descended into the scrotum (PND15 and older), the incubation temperature of the relevant samples must be decreased to 32°C, the average temperature measured in the scrotal area (between 30.1–34°C) of mice (Li et al., 2013). If the incubation temperature for adult samples is adjusted to 37°C, it induces the expression of heat-shock proteins, initiates oxidative stress, which leads to increased apoptosis of primary spermatocytes impacting the expression of physiologically important genes in SCs (Jegou et al., 1983; Seethalakshmi and Steinberger, 1983; Aldahhan et al., 2019, 2021).

Time. The preparation of a single cell suspension from ST should be completed in less than 2 h, which includes cell incubation and antibody staining. The ensuing FACS sort is usually completed within 60 min. After this time, there is not only a diminishment of primary spermatocytes (Gaysinskaya et al., 2014; Rodriguez-Casuriaga and Geisinger, 2021) but also a decrease in potential changes in the gene expression profile of SCs.

Tenderness. Samples should be handled gently, neither shaken nor vortexed at any point during the procedure. The incubation of cell samples at either 37°C or 32°C must be conducted without agitation. The primary reason being that SCs with their large cytoplasmic volume and columnal shape are vulnerable to shearing forces.

We adapted a single cell suspension preparation method to SC isolation, the utility of which has been proven over time, highly cited in the literature, and routinely used in laboratories that study spermatogenesis but mostly targeted non-SC cell types (Bellve et al., 1977b). While speed, cell viability, and accuracy in targeting SCs are the major advantages of this protocol, it can also be used for isolation and FACS-sorting of any particular subset of GCs residing in STs or a combinatorial sorting of these cell subsets. Importantly, when our protocol was combined with FSHr^+^/Ho Blue FACS profiling of SCs, it achieved a 10–100-fold diminishment in the level of contaminating transcripts of the main types of GC-specific gene markers. This method also removed all contaminating somatic cells such as peritubular myoid cells and SSCs, the presence of which was a major disadvantage of adherent strategies for SCs enrichment from samples (Bhushan et al., 2016; Lakpour et al., 2017).

### Advantages and potential pitfalls of our method

This novel approach for the isolation of a pure population of SCs certainly provides many advantages but also possesses inherent limitations and potential pitfalls. The analysis of bulk-RNAseq from FACS-sorted SCs was comparable to the results obtained by Zimmermann et al. not only in terms of the expression of marker genes in SCs but also in regards to a clear distinction in specific gene expression characteristics observed in the juvenile and adult developmental stages of SCs. The advantage of this method over previously published protocols is the ability to efficiently isolate SCs without using transgenic animals with fluorescently tagged SCs. Moreover, our method of SC isolation is not limited to a specific mouse strain, in this case Sox9-eGFP which is on C57Bl6/129Sv mixed genetic background. In addition, this approach can be the method of choice for other molecular applications such as epigenetic studies or single-cell RNA analysis of SCs from distinct developmental stages. Nevertheless, due to the strong intrinsic phagocytic activity of SCs during spermatogenesis and spermiation, we observed that GC transcripts are an inseparable part of the cytoplasmic RNA pool of SCs (Kerr and de Kretser, 1974; Russell et al., 1989; Sakai and Yamashina, 1989). This finding is in a full agreement with the observations of Zimmermann et al., who adapted a subtractive strategy to eliminate transcripts which exhibit an obvious meiotic and post-meiotic germline origin. Our strategy was to decrease GC-specific transcript contamination in SC samples by FACS-sorting SCs according to precisely measured 2C content and FSHr surface staining.

## Conclusions and perspective

The production of vital sperm during meiosis requires coordinated interplay between SSCs, GCs, and SCs, which make up the spermatogenic niche (Kostereva and Hofmann, 2008; Xi et al., 2022). Since SCs interact directly with GCs and are instrumental in providing morphological and nutritional support for spermatogenesis, their dysfunction is often linked to the failure of spermatogenesis (Flanagan et al., 1991; Hiort et al., 2000; Ferlin et al., 2006; Bashamboo et al., 2010). In the past, scientists have attempted to understand the relationship between somatic and GCs in the STs. We believe, that this novel method will accelerate new advances in the field of SC physiology and their essential role in spermatogenesis.

## Data Availability

The datasets presented in this study can be found in online repositories. The names of the repository/repositories and accession number(s) can be found below: ArrayExpress accession number E-MTAB-11895.
