# Supplementary material for: Two complementary approaches for efficient isolation of Sertoli cells for transcriptomic analysis
Source: Front Cell Dev Biol. 2022 Sep 6;10:972017. doi: 10.3389/fcell.2022.972017 (PMC9495933; doi:10.3389/fcell.2022.972017)
Supplement: Supplementary file 1 [file DataSheet1.PDF]

Supplementary Material

Figure S1

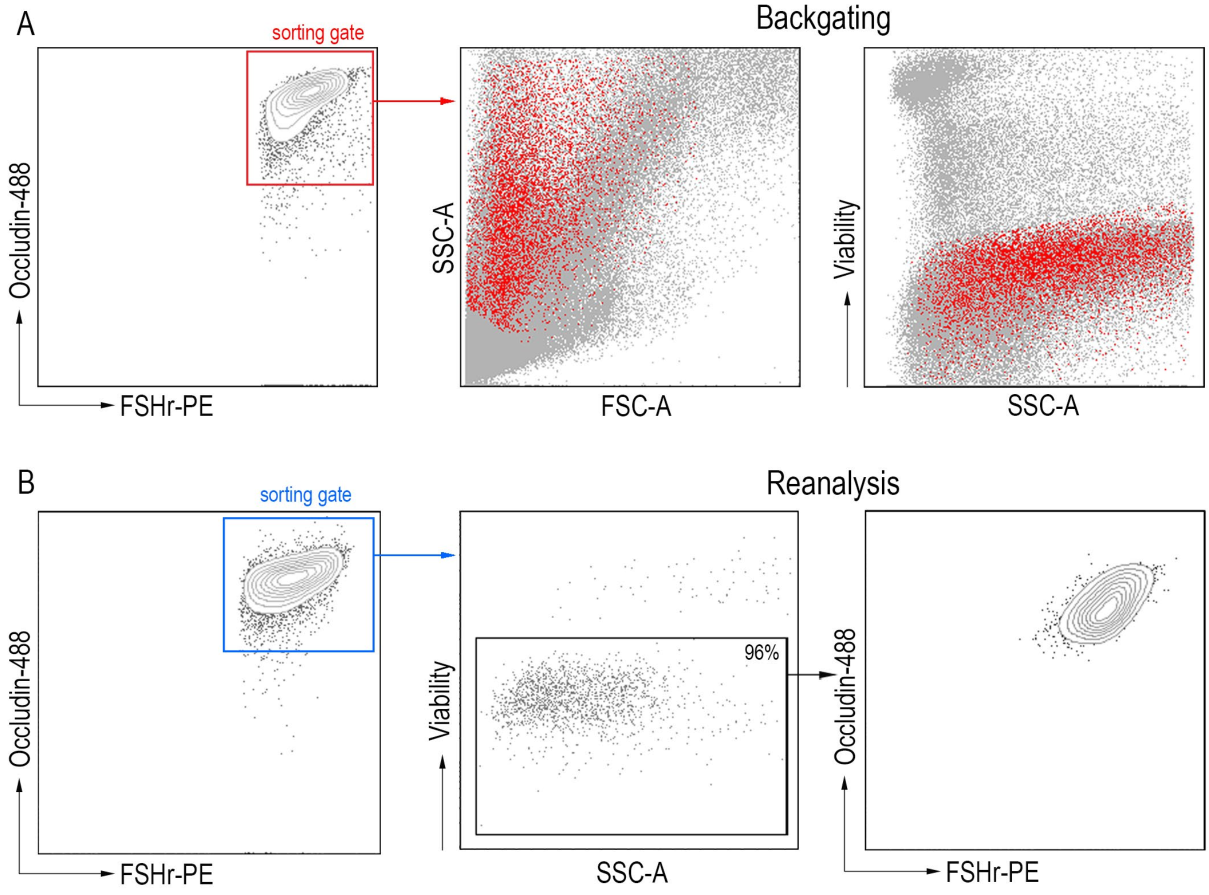

Supplementary Figure 1. (A) Back gating of the FSHr-PE/Occludin-488 double positive cell population showing the forward versus side scatters and side scatters versus viability (Hoechst 33258). **(B)** Post-sort reanalysis of Sertoli cells showed their high vitality (96%) with all cells positive for sort markers.

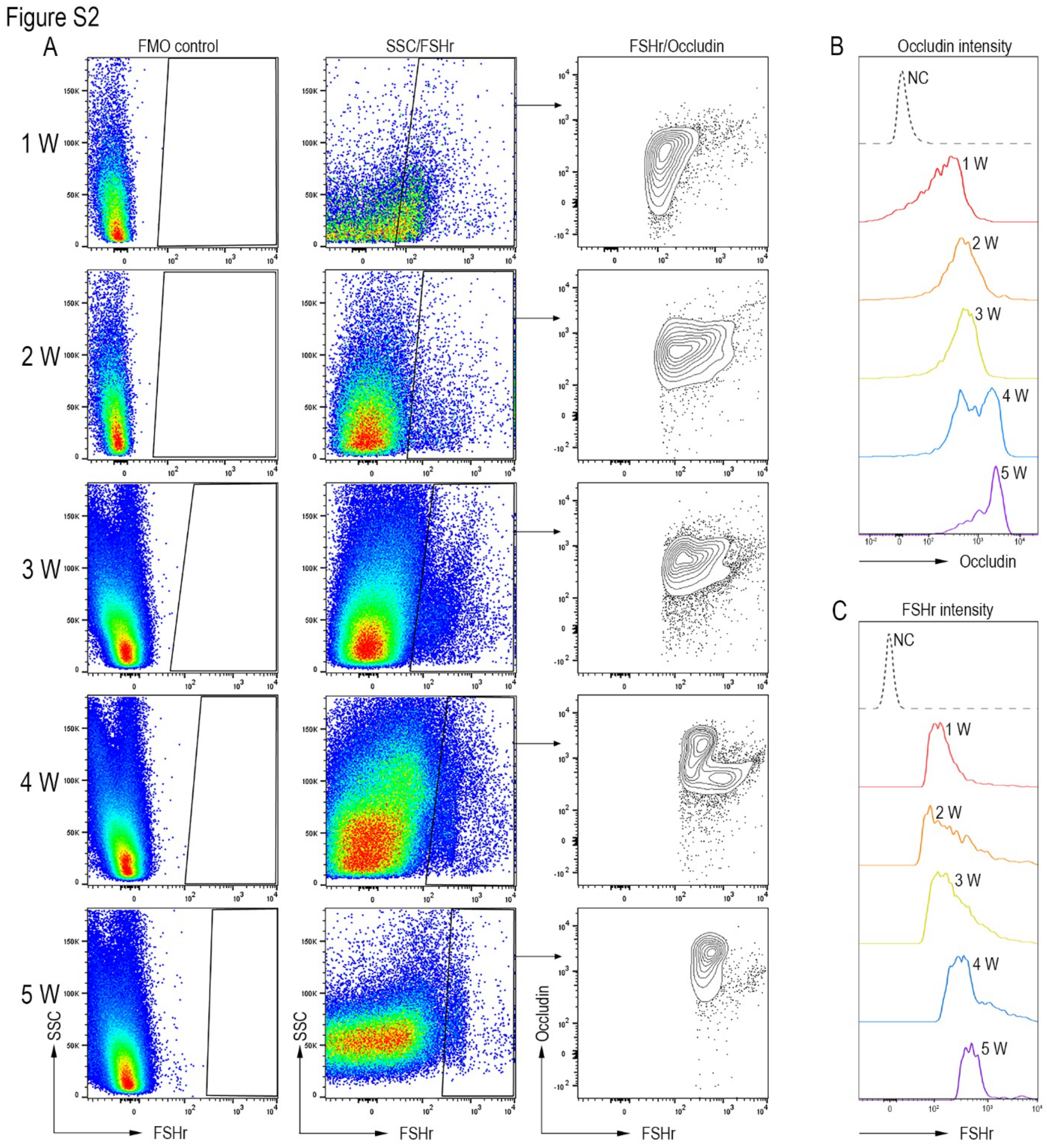

Supplementary Figure 2. (A) Flow cytometric analysis of SCs from 1- to 5-week-old males confirmed the surface expression of FSHr and Occludin. The FSHr positive gate was established according to the FMO control. **(B)** Occludin intensity histograms from (A) show a gradual increase in Occludin signal intensity, which is more intense in SCs from sexually maturing 4-5 week-old males. **(C)** FSHr intensity histograms from (A) show a relatively stable expression of surface FSHr on SCs which is more enhanced in 4- to 5-week-old males. NC = negative control.

Figure S3

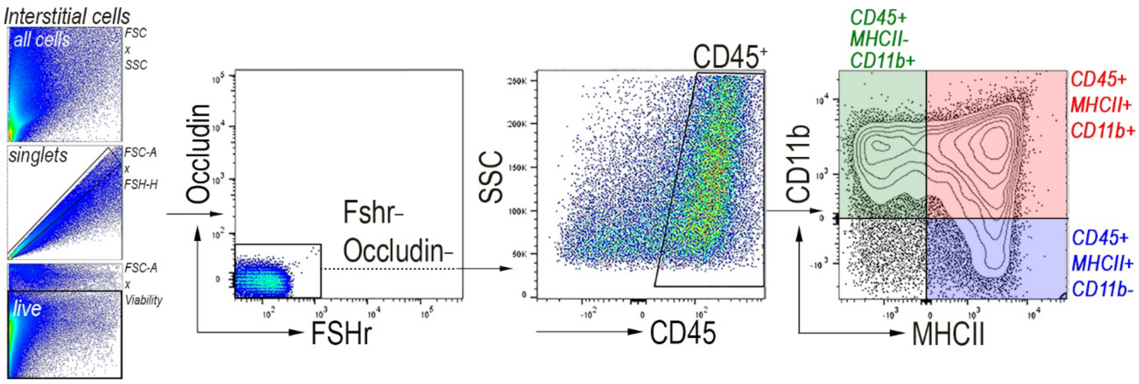

Supplementary Figure 3. The population of interstitial cells (three panels on the far left) is completely negative for FSHr and Occludin surface markers (the panel second from the left). In contrast, immune cells (CD45+ gate) which are present in the interstitial space along with nonimmune cells (CD45–), were also positive for additional immune markers, CD11b and MHCII (right panel). Their combined immune phenotypes are color coded.

Figure S4

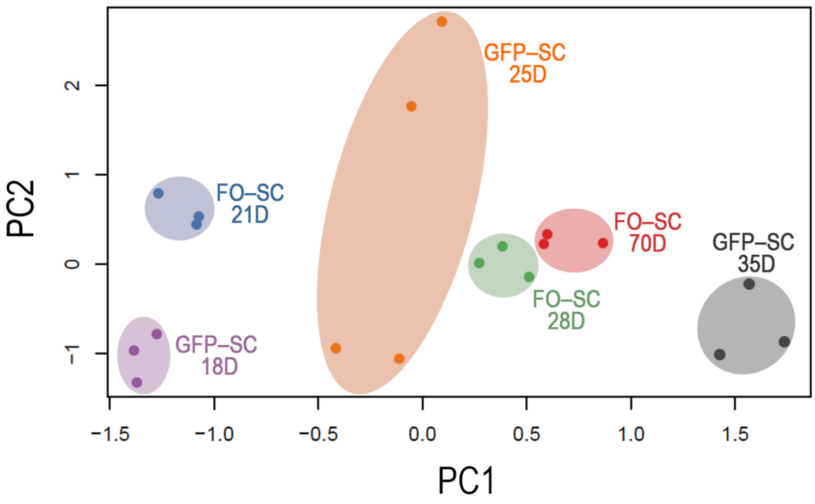

Supplementary Figure 4. PCA analysis of the top 25% of the most variable genes shared between the datasets analysed in this study, i.e. Zimmerman's (Zimmermann et al., 2015) and our samples. We observed that even though methodological differences between these two studies are apparent, PCA analysis of the samples analysed revealed clustering in respect to the developmental time point (reflected by PC1) at which the Sertoli cells were isolated and analysed.

KRB	
**Chemical**	**Final concentration**
NaCl	135 mM
KCl	5 mM
K_2HPO_4	0.4 mM
Glucose	5.5 mM
HEPES	20 mM
$MgSO_4$	1 mM
**EKRB (KRB + following)**	
$CaCl_2$	1 mM
$NaHCO_3$	17 mM
Sodium Lactate	0.5 mg/ml
Sodium Pyruvate	1 mM

Supplementary Chart 1. KRB buffer may be prepared in advance, aliquoted and stored at -20°C. EKRB should be prepared by adding individual chemical components to KRB on the day of experimentation.

Primer name	Forward	Reverse
**Wt1**	GAGAATCCGCAGGATCGCAG	TGAACTGGCCCGAGAAGTG
**Amh**	GCAGTTGCTAGTCCTACATC	TCATCCGCGTGAAACAGCG
**Top2a**	TACAGTGCTCAACCTCTGACG	GGGATCTCGTGTTGGGAAGG
**Prdm9**	ATCGAACCTGCCGGGTTATC	ATAATAGTGCCCACCTCTTCCT
**Sycp1**	ACCGTTGGACAACGATTGCT	ATCCATTGCAAGTAAAAGCAACA
**Sycp3**	GAATGTGTTGCAGCAGTGGG	TGCCATCTCTTGCTGCTGAG
**Acrv1**	AGACCAACAAGCTTCGGTTCA	AGTGCTCACCTGCAGAATGT
**Catsper3**	CTTCAGTTTGGCCACGGTTG	CCCAGCTACGGCTACCTCTA
**Prm3**	GTCCTCCATGAAGAAGCTCGT	TGAATGTCCTCTGGCGTGG
**Izumo2**	AAATGAAAGCCTGTGACCACAAAA	CTGCCCATCCTCAAAGCAGTA

Supplementary Chart 2. Primers used for qPCR. All primers anneal at 60°C.
